# Approaches to Potentiated Neuroprotective Treatment in the Rodent Model of Ischemic Optic Neuropathy

**DOI:** 10.3390/cells10061440

**Published:** 2021-06-09

**Authors:** Zara Mehrabian, Yan Guo, Neil R. Miller, Amanda D. Henderson, Steven Roth, Steven L. Bernstein

**Affiliations:** 1 Department of Ophthalmology and Visual Sciences, School of Medicine, University of Maryland at Baltimore (UMB), 10 S. Pine St., MSTF Room 5-77B, Baltimore, MD 21201, USA; Zmehrabyan@som.umaryland.edu (Z.M.); yanguo@som.umaryland.edu (Y.G.); 2 Division of Neuro-Ophthalmology, Wilmer Eye Institute, School of Medicine, Johns Hopkins University, 600 N. Wolfe St., Wilmer 233, Baltimore, MD 21287, USA; nrmiller@jhmi.edu (N.R.M.); ahende@jhmi.edu (A.D.H.); 3 Department of Anesthesiology, College of Medicine, University of Illinois, Chicago, IL 20212, USA; rothgas@uic.edu

**Keywords:** nonarteritic anterior ischemic optic neuropathy (NAION), animal model, ischemia, rodent, optic nerve, prostaglandin, PGJ_2_, retinal ganglion cell, neuroprotection, inflammation, gene expression

## Abstract

Nonarteritic anterior ischemic optic neuropathy (NAION) commonly causes sudden optic nerve (ON)-related vision loss. The rodent NAION model (rAION) closely resembles NAION in presentation and physiological responses. We identified early rAION-associated optic nerve head (ONH) inflammatory gene expression responses and the anti-inflammatory prostaglandin PGJ_2_’s effects on those responses. We hypothesized that blocking pro-inflammatory prostaglandin (PGE_2_) production by inhibiting monoacylglycerol lipase or cyclooxygenase activity and co-administering PGJ_2_ would potentiate RGC survival following ischemic neuropathy. Deep sequencing was performed on vehicle- and PGJ_2_-treated ONHs 3d post-rAION induction. Results were compared against responses from a retinal ischemia model. Animals were treated with PGJ_2_ and MAGL inhibitor KML29, or PGJ_2_ + COX inhibitor meloxicam. RGC survival was quantified by stereology. Tissue PG levels were quantified by ELISA. Gene expression was confirmed by qPCR. PGJ_2_ treatment nonselectively reduced inflammatory gene expression post-rAION. KML29 did not reduce PGE_2_ 1d post-induction and KML29 alone increased RGC loss after rAION. Combined treatments did not improve ONH edema and RGC survival better than reported with PGJ_2_ alone. KML29′s failure to suppress PGE_2_ ocular synthesis, despite its purported effects in other CNS tissues may result from alternative PG synthesis pathways. Neither KML29 nor meloxicam treatment significantly improved RGC survival compared with vehicle. While exogenous PGJ_2_ has been shown to be neuroprotective, treatments combining PGJ_2_ with these PG synthesis inhibitors do not enhance PGJ_2_’s neuroprotection.

## 1. Introduction

Nonarteritic anterior ischemic optic neuropathy (NAION) is an ischemic lesion of the anterior portion of the optic nerve (the optic nerve head; ONH), and the most common cause of sudden optic nerve-related vision loss in individuals over the age of 50 [1]. Most NAION cases are hypothesized to occur following initial vascular dysregulation, which results in capillary decompensation with development of edema in the restricted ONH space. The subsequent edema generates an ONH-compartment syndrome with vascular compression and axonal ischemia [2,3], which ultimately causes a loss of retinal ganglion cell (RGC) neurons and their axons. Currently, no clinically proven effective treatments exist for this disorder [4], although multiple approaches have been tried, including steroids, memantine, vascular endothelial growth factor inhibitors, and more recently a phase 2/3 clinical trial using siRNA against caspase 2 (https://clinicaltrials.gov/ct2/show/NCT02341560 (accessed on 18 November 2020)).

Following ischemic stress, CNS membrane lipids are metabolized to arachidonic acid (AA), generated either by phospholipase A_2_ (PLA_2_), or by degradation of the endocannabinoid 2-arachidonylglycerol via monoacylglycerol lipase (MAGL). Although phospholipase A_2_ (PLA_2_) was originally proposed as the main generator of prostaglandins (PGs) [5], MAGL is currently believed responsible for the majority of the AA precursor pool in brain [6]. AA is subsequently metabolized by cyclooxygenase 1 and 2 (COX1/COX2) to PGH_2_ and then to the various PGs. We previously demonstrated that pharmacological doses of the *anti*-inflammatory prostaglandin J_2_ (PGJ_2_), which is nonenzymatically derived from PGD_2_, neuroprotect in the rodent and primate models of NAION (rAION and pNAION) [7,8], and that this effect appears to occur by reducing post-rAION inflammation. PGJ_2,_ the *pro*-inflammatory prostaglandin E_2_ (PGE_2_) and other prostaglandins are synthesized following CNS ischemia [9,10]. PGE_2_ binding to specific receptor subtypes is believed to enhance injury and post-stroke functional deficits [11]. We hypothesized that we could potentiate PGJ_2_’s neuroprotective effect by simultaneously suppressing PGE_2_ synthesis by MAGL inhibition. MAGL inhibitors by themselves have been reported to be neuroprotective in mouse models of Alzheimer’s disease, Parkinson’s disease and stroke [5,12,13].

PG action can be exerted via their specific receptors, which can have varied effects on inflammation and neurodegeneration. For example, PGE_2_ receptors 1 and 3 are associated with neurotoxicity and inflammation [11,14,15,16]. PGJ_2_ is unique in that it is nonenzymatically derived from PGD_2_, and while PGD_2_ functions by binding to PGD_2_ receptors (DP1 and DP2), PGJ_2_ exerts its action by binding to peroxisomal proliferator-activated receptor *gamma* receptor (PPARγ) or to the nuclear factor kappa-B kinase (IKKB), which inhibits NFkB activation and inflammation [17]. PGJ_2_ can also induce ischemia-protective molecules such as heme oxygenase-1, which exert anti-inflammatory actions including generating the gasotransmitter carbon monoxide (CO) [18]. Our current report had three aims: (1) Identify the eicosanoid-associated expressed genes in the naïve ONH and both eicosanoid-associated and inflammation-related genes in the ONH shortly (3d) after induction of rAION to gain a better understanding of inflammation-related genes during ON ischemia and the in vivo response to PGJ_2_ administration. (2) Compare the relative inflammatory expression pattern of ischemia of the ONH with that of the retina to determine whether there are differences in the inflammatory responses to ischemia in the two tissues, which was possible because a previous study evaluated retinal responses to ischemia and the effects of ischemic preconditioning [19]. (3) Determine whether PGJ_2_’s RGC-neuroprotective effect could be potentiated by concurrent suppression of PGE_2_ by either MAGL inhibition or downstream PG suppression using a COX1/2 inhibitor (meloxicam). Importantly, and unlike all previous ON (and many higher CNS) neuroprotection studies, we directly evaluated PGE_2_ levels in the presence and absence of the MAGL inhibitor.

## 2. Materials and Methods

All animal protocols were approved by the UMB institutional animal care and use committee (ACUO), Project Protocol number 0717003. 150 Male Sprague Dawley rats (200–250 g) were utilized in this study.

### 2.1. Anesthesia and rAION Induction

Animals were anesthetized with an intraperitoneal mixture of Ketamine and Xylazine (80 mg/4 mg/kg), and kept on warming pads during anesthesia and recovery, to minimize core body temperature changes. The pupils were dilated with topical 1% cyclopentolate-2.5% phenylephrine, and corneas were topically anesthetized with 0.5% proparacaine. We placed a planoconvex contact lens enabling visualization of the retina and optic nerve. To minimize off-nerve effects that can occur with nondedicated plastics, and to enable easy replication of this method by other labs, we worked to generate a readily available commercially manufactured rat fundus contact lens (Micro-R 2.50/7.00 Calibration Test Piece; Nissel and Cantor, Northampton, UK). Animals were rAION induced using intravenous injection of 1 mL/kg of a 2.5 mM solution of rose bengal in Dulbecco’s phosphate-buffered saline (D-PBS; pH 7.4) administered via tail vein. Thirty seconds post-injection, the intraocular portion of the optic nerve was illuminated for 10 s with a clinical 532 nM wavelength laser (Oculight GLx; Iridex, Mountain View CA, USA), spot size 500 um diameter and 50 mW power. Animals were allowed to recover, and utilized for individual analyses. The same contact lens was used for subsequent direct slit-lamp fundus examination and spectral domain optical coherence tomography (SD-OCT) analyses.

### 2.2. Deep Sequencing of the ONH

A total of 15 animals were used for this part of the study. We isolated total RNA from the ONHs of 5 individual naïve animals (ONHs from both eyes of each animal pooled as a single sample). We evaluated a single pooled RNA sample from 3d post-rAION animals (*n* = 5 ONHs; 11 sec induction; see below) and a single pooled RNA sample (*n* = 5 ONHs) from 3d post-rAION animals that were treated with PGJ_2_ immediately after induction.

### 2.3. Combinatorial Treatments

A total of 55 animals were used for this part of the study. For the RGC protection analyses, we performed a power analysis for the combinatorial (KML29 + PGJ_2_) vs. vehicle comparison. We used 16 animals for KML29 + PGJ_2_ administration. Ten animals received vehicle. Six animals received PGJ_2_ alone to confirm previous effects. Thirteen animals received the MAGL inhibitor KML29 alone. Significance for the MAGL-combinatorial study was set at *p* < 0.05. We utilized a two-tailed t-test comparing KML29 + PGJ_2_ vs. either KML29 or vehicle alone. Because we were also interested in the neuroprotective role of COX inhibition downstream of MAGL activity, we also performed a preliminary evaluation utilizing a smaller number of animals with a COX1/2 inhibitor meloxicam (Metacam; Boehringer-Ingelheim, Ingelheim Germany). Four animals received the COX inhibitor meloxicam alone, whereas 6 animals received PGJ_2_ + meloxicam. The smaller numbers of animals for the COX inhibitor secondary analyses necessitated use of a Mann-Whitney nonparametric statistical test. A power calculation was performed and the number of animals used conformed to these prior calculations.

### 2.4. Treatment Regimens

The highly selective MAGL inhibitor KML29 was obtained from Pfizer corporation under their pure compound grant program (CTPGrants@Pfizer.com (accessed on 10 December 2018) [20]. KML29 is more potent in rats than JZL184 [21], blocking 99% of MAGL activity in vitro and a single dose in mice reduces CNS PGE_2_ levels by >75% when given at a dose of 10 mg/kg [22]. Interestingly, KML29 does not suppress PGD_2_ synthesis in the spinal cord [22]. We directly evaluated KML29′s in vivo effect on ocular tissue prostaglandin synthesis by enzyme-linked immunosorbent assay (ELISA) (see paragraph below). KML29 was suspended in 10% *v/v* ETOH, 10% *v/v* Kolliphor EL (Sigma-Aldrich; St. Louis, MO, USA) and 80% *v/v* D-PBS (pH 7.4). The mixture was sonicated for 90 s at 40 W, then administered subcutaneously (SC). Meloxicam (1 mg/kg) was administered SC and PGJ_2_ was administered IV. All treatments were begun immediately after rAION induction. Second doses of KML29, meloxicam or vehicle were administered 12 h post-induction. 15 deoxy, delta 12,14 PGJ_2_ was purchased from Cayman chemicals, and resuspended in 20% ethanol-D-PBS at a concentration of 100 ug/mL. PGJ_2_ was administered as a single IV dose immediately post-induction at a concentration of 100 ug/kg. Thus, animals receiving combinatorial treatments received two injections immediately post-induction—1 IV and the second SC, followed 12 h later by SC only. Animals tolerated this approach well.

### 2.5. Retinal Examination and Intraocular ON Edema Quantification via SD-OCT

**Figure 1:**
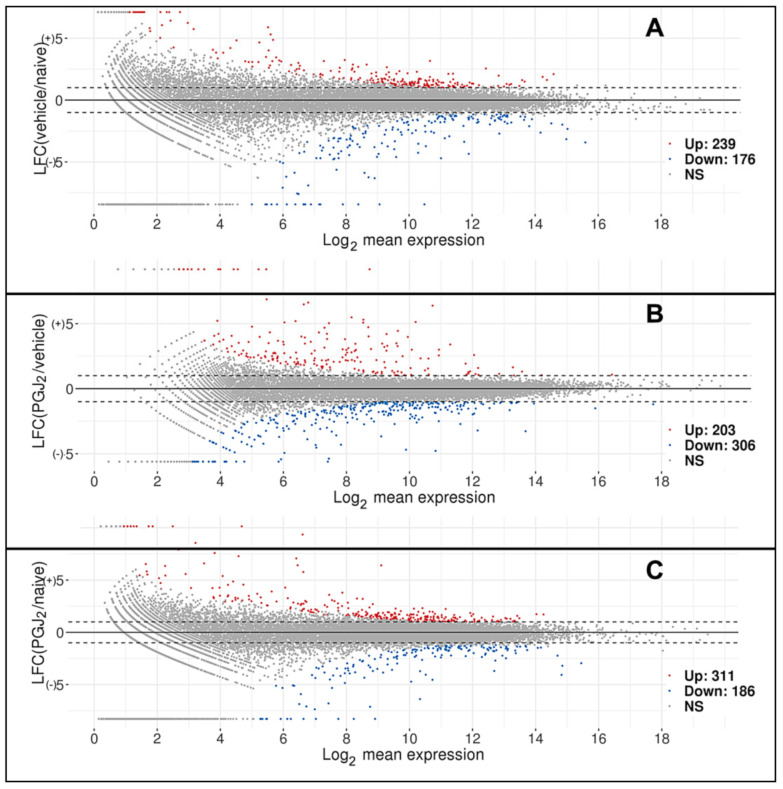
MA plots comparing differences between two samples and mean expression. Plots display the relationship between log-fold expression change (LFC: M) between the two samples and average expression. Log_2_ mean expression: (**A**) Comparison between mean naïve and 3d post-rAION-induced, vehicle-treated ONH. (**B**) Comparison between rAION-induced animals treated with PGJ_2_ and vehicle. (**C**) Comparison between naïve and PGJ_2_-rAION-induced animals. Individual genes are shown in Supplemental Table S1. False discovery rate (FDR) was set at ≤0.05 and log_2_-fold change (LFC; on ordinate scale) ≥ +/1). Log_2_ mean expressions for the genes are shown on the abscissa. Dashed lines indicate the LFC cutoff values of +/1. Points in gray indicate those genes that are not significantly expressed. Points colored in red indicate the upregulated genes, while points in blue indicate the downregulated genes.

Two days post-induction, eyes were examined and quantified for maximal ONH edema. Animals were anesthetized, their eyes re-dilated and corneae topically anesthetized. A plano-convex contact lens was placed for direct retinal visualization and color photos via slit lamp. Following retinal visualization, both eyes of each animal were imaged using a Heidelberg SD-OCT instrument (Heidelberg Corp, Heidelberg, Germany), with attached 25 diopter rodent eye correcting lens + contact lens, for both *en face* and intraretinal cross-section analysis, imaged at 15 degrees (10 section analysis). The contact lens enables effective retinal cross-sectional visualization, which cannot be obtained by correcting lens alone. The intraocular ON diameter was measured by measuring the distance between the inner nuclear layer (INL) images that are separated by the ON (in microns, using the included imaging tool in the Heidelberg instrument for each intraretinal cross-sectional image (See Figure 1B), and then calculating the mean intraretinal INL–INL distance from the diameter of the three contiguous INL–INL images with the greatest distance.

### 2.6. Interrogation by Expression Profiling: RNA Isolation, ONH Sequencing and Analysis

The ONH segment (the most anterior 1 mm of the ON is a transitional region between the retina and myelinated optic nerve and does not resemble either tissue completely. ONH was dissected from the globe 1d post-induction following retinal and scleral wall removal, separated from the distal ON by section at 1 mm distal to the globe, and fast-frozen on dry ice. An additional ON segment was isolated at least 2 mm posterior to the globe to avoid ONH contamination, for independent ON analysis. Because ONH contains considerable connective tissue in addition to neural elements, we optimized RNA isolation using a two-step technique that we found enhanced recovery. Total RNA was initially isolated using guanadinium thiocyanate-phenol reagent (RNABee; Tel-Test; Friendswood, TX, USA), followed by resuspension in guanidinium hydrochloride reagent and subsequent column purification (Qiaprep micro RNA kit (Qiagen Corp; Hilden, Germany).

Naïve ONH samples utilized total RNA from individual ONHs for independent analyses via deep sequencing. Post-stroke gene expression analysis was performed in two ways: (1) Total RNA was pooled from a group of 5 rAION-induced ONHs from animals treated with PGJ_2_ and compared with rAION-induced ONHs from animals treated with vehicle. (2) Individual ONH-qPCR-based analyses from samples collected at 3 days post-induction (maximal inflammation). Total RNA quality was evaluated using an Agilent 2100 Bioanalyzer System Total RNA Pico Series, Santa Clara, CA prior to use. Following linker addition, cRNA probes were generated for Illumina sequencing, and samples were interrogated to a depth of 150,000,000 nucleotides/lane. Sequence data for ONH were compared using Deseq2-normalized (log_2_-fold relative levels) expressed in ONH RNA from individual naïve, mean naïve, rAION-vehicle-induced and rAION-PGJ_2_-treated animals, using a cutoff of <3.4. Normalization is based on Trimmed Mean Values (TMM) and normalized by EdgeR-cpm function with a cutoff of 2. Naïve retina (retina-N) and ischemic retina (retina-Isch) data are from [19] and expressed in counts per million (CPM) without normalization. Additional analysis was performed using Ingenuity pathway analysis software (Qiagen; Hilden, Germany). Inflammatory gene pathways were analyzed, as were PG synthesis and associated pathways.

### 2.7. Quantitative Real-Time PCR (qPCR) Analysis

qPCR analyses utilized total RNA preps from three individual samples for each analyzed condition (naïve, vehicle-injected, PGJ_2_-treated and PGJ_2_ + KML29). Following predigestion with DNase 1 to remove genomic contamination, first strand cDNA was prepared via random priming (Super Script VILO kit, Invitrogen, CA, USA). Single-stranded cDNA substrates were then utilized for candidate gene qPCR analysis, using gene-specific primer pairs optimized for a Tm of 60–61 °C, and product lengths of 100–150 bp. Cyclophilin B and β-actin were used as internal reference standards. qPCR results were converted to ddCT format. Gene primers are shown in Table 1.

### 2.8. Enzyme-Linked Immunosorbent Assay (ELISA) of Ocular Tissues

To confirm effects of MAGL inhibitory treatment, we performed ELISA on retina, ONH and ON of six animals induced with rAION and treated with either vehicle or KML29 for 24 h. The time was selected because drugs previously shown to be effective in the rAION model must be administered within 1 day of induction. Two animals were used per treatment condition (total 6 rats for three conditions), and one eye of each animal was rAION induced at time 0. We obtained retinae, ONH and ONs from each animal.

Tissues were dissected on ice, flash frozen on dry ice, and stored at −80 °C until extraction. The tissues were homogenized in ice-cold D-PBS buffer containing 0.05% Triton X 100, 10 mM EDTA, pH 8.0 and Halt protease -phosphatase cocktail (#78441; Thermo Fisher Scientific; Waltham, MA) using sonication on ice with 3–30 s bursts of 40 W energy. Samples were incubated on ice for 15 min to release prostaglandins and spun down (3000× *g* for 3 min) to remove cell debris; 10 uL aliquots were taken for protein concentration estimation by Lowry (Bio-Rad; Hercules, CA, USA). Rest of the samples were adjusted to pH 3.5 with 2N hydrochloric acid and applied to C18 reverse phase columns cartridges (#221-0010 Biotage; Uppsala, Sweden) pretreated with ethanol and water per manufacturer’s instructions. Loaded samples were pushed with a plunger, rinsed subsequently with water, ethanol and hexane and eluted with ethyl acetate. Eluates were evaporated under stream of nitrogen and resuspended in assay buffer. ELISA (for PGE_2_ and PGJ_2_) was performed following manufacturer’s protocol Enzo (ADI-900001 and ADI-900-023, respectively) and optical density readings were taken at 405 nm with the correction at 590 nm on Varioskan LUX (Thermo Fisher) microplate reader.

### 2.9. RGC Stereology

Thirty days post-induction, animals were euthanized, and eye and ONs isolated and post-fixed in 4% paraformaldehyde-PBS pH 7.4 (PF-PBS). PF-PBS-fixed retinae were isolated, and RGCs immunostained using goat polyclonal anti-Brn3a antibody (sc-31984; Santa Cruz Chemicals; Santa Cruz, CA, USA), and then visualized with donkey anti-goat Cy3-conjugated IgG (Jackson Immunoresearch; West Grove, PA, USA) and stereologically counted as previously described [23], using a Nikon Eclipse E800 fluorescent microscope (Nikon, Melville, NY, USA) with motorized stage, driven by a stereological imaging package (StereoInvestigator, Ver 10.0; Microbrightfield Bioscience (MBF), Williston, VT, USA). Stereological analysis was performed using the Stereo Investigator 10 package, with counts in each eye greater than that required by the Schmitz-Hof equation for statistical validity.

## 3. Results

### 3.1. Deep Sequencing Reveals ONH Emphasis on Prostaglandin Synthesis and Function

A total of 22,220 genes were identified by DeSeq 2 after normalization with a minimum fold value >3.66. The data are shown in Supplemental Table S1. A comparison of vehicle (rAION) with naïve (uninduced) sequence identified 239 genes that were upregulated and 176 genes that were downregulated, compared with naïve (uninduced) (MA plot; Figure 1A). We also performed a comparison of expression between rAION-induced, PGJ_2_- and vehicle-treated ONH, which revealed that 203 genes were upregulated and 306 genes downregulated between the two groups (Figure 1B), and between PGJ_2_-treated and naïve (uninduced) groups (Figure 1C). The majority of genes differentially expressed in group 1B (PGJ_2_ and vehicle treated) were membrane-related proteins such as myelin protein zero and fucose mutarotase, although a number of ribosomal proteins and reactive oxygen-associated genes were also identifiable. These genes are shown in Supplemental Table S1.

We compared the relative expression of the group of identified prostaglandin-related synthetic genes and prostaglandin receptors in these different treatment groups (Table 2). To evaluate relative differences in retina and ONH expression in naïve and ischemic conditions, we also included gene expression data obtained from retinal deep sequencing data, from a previous study using Sprague Dawley (SD) rats that compared expression both from naïve retina (sham treated) and retina that was subjected to induced ischemia from increased intraocular pressure for 55 min (retina + ischemia + sham post-conditioning) at one day post-induction [19]. These data are shown in the last three columns; the ratio of retina expression for these genes in the two conditions is shown in the last column (Table 2).

The CNS form of prostaglandin D_2_ synthase (Ptgds) (which gives rise to PGJ_2_) and the cytosolic/constitutive form of PGE synthase (Ptges3) were expressed in ONH at 17.10 and 7.83, respectively (Table 2), while the housekeeping genes β-actin (Actb) and cyclophilin B (Ppib) were expressed at 17.08 and 13.30 (Table 1; housekeeping gene expression is shown in Table 3). There was no expression of either prostaglandin F (Ptgfs) or prostacyclin (Ptgis) synthases, suggesting that PGD_2_, and its nonenzymatically derived metabolite PGJ_2_, and PGE_2_ are the major prostaglandin forms likely to be generated in the ONH prior to other induction.

We also evaluated the different arachidonic acid (AA) synthetic genes. Both MAGL/MGLL and PLA_2_ gene isoforms were normalized and compared in naïve, vehicle-induced and PGJ_2_-treated ONH (Table 3). MAGL/MGLL is expressed in the naïve ONH at mean levels ~10.0 and this does not significantly increase after rAION induction. A total of 17 individual PLA_2_ isoforms were co-expressed along with MGLL/MAGL (Table 3). A number of PLA_2_ isoforms mRNAs were found at levels considerably higher (6–20 fold) than that seen for MAGL expression (Table 3). For example, MGLL/MAGL was expressed at a mean of 9.87, while cytosolically expressed PLA_2_ group 7 (Pla2g7) was expressed at a mean of 14.09, or approximately 16-fold greater than that for MAGL. Two additional PLA_2_ isoforms were expressed at higher levels than MGLL (Pla2g16 and Pla2g6) (Table 3).

Relative expressions of MGLL/MAGL and PLA2 isoforms in the ONH and retina were markedly different: six PLA_2_ isoforms were expressed at levels in the ONH far exceeding anything in retina (Table 3), with one cytosolic PLA_2_ isoform (Pla2g7) expressed at levels considerably greater in the ONH than other isoforms. Six PLA_2_ isoforms were expressed in the ONH at greater levels than MGLL/MAGL, but in the retina, only one isoform (Pla2g16) was expressed at levels greater than MGLL/MAGL, while the other PLA_2_ isoforms were found at considerably lower levels (Table 3). Both the specific cytosolic PLA_2_ isoforms expressed at the highest level are different in ONH and retina, and the ONH expresses considerably higher levels of a number of PLA_2_ mRNAs than it does MGLL/MAGL, suggesting that the ONH may be less responsive to MGLL/MAGL suppression than the retina.

### 3.2. rAION-Associated Inflammatory Response: PGJ_2_ Exerts Action Post-rAION by Nonselective (M1/M2) Inflammatory Suppression

**Figure 2:**
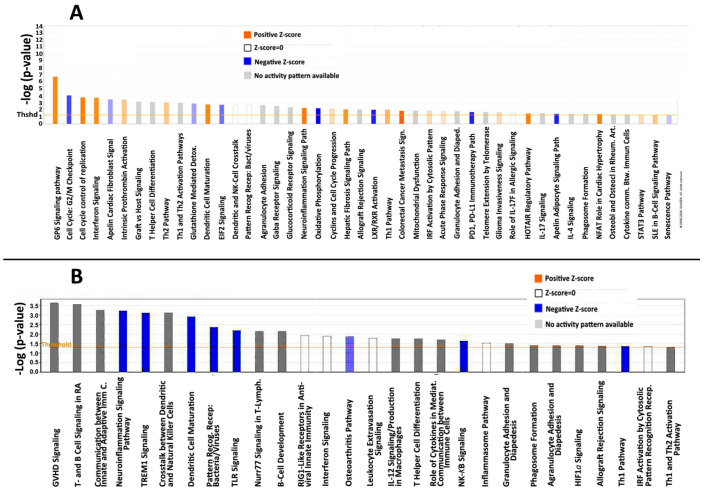
Ingenuity pathway analysis of rAION responses. Pathways evaluated by Ingenuity software package (http://www.ingenuity.com (accessed on 29 August 2020)). (**A**) Comparison between naïve and rAION-induced, vehicle-injected ONHs. B) Comparison between rAION-induced, vehicle-injected and rAION-induced, PGJ_2_-treated animals. The log *p*-value response threshold for significance is set at 1.5 fold. Orange signal represents upregulation; blue signal represents downregulation; and white represents equivalent expression levels of pathway members altered both up- and downregulated, while grey signal represents uninterpretable (inconsistent) pathway response. In (**A**), comparison between naïve and vehicle gene sets reveals strong upregulation of interferon-associated pathway signaling, neuroinflammation and dendritic cell maturation pathway signaling, with moderate upregulation of components associated with both the Th1 and Th2 pathways. There is strong downregulation of the G2/M cell cycle checkpoint (=mitotic upregulation), liver-X and retinoic acid (LXR/RXR) pathway components. LXRs are inhibitors of inflammation. In (**B**), following PGJ_2_ treatment after rAION, there is suppression of the neuroinflammation, TLR and TREM-macrophage and -neutrophil signaling pathways, as well as suppression of leukocyte extravasation and Th1 signaling, but not Th2. The decrease in NF-κB signaling confirms PGJ_2_’s known role in suppression of this system.

In addition to astrocyte-associated intrinsic responses, extrinsic macrophages have been shown to extensively infiltrate the ONH in the first three days post-induction [24]. Since extrinsic macrophages can be activated along either Th1/neurodegenerative or Th2/neuroprotective pathways [25], we performed Ingenuity pathway analyses comparing the gene responses of (1) naïve vs. rAION-induced vehicle-injected ONHs (inflammatory pathway analysis) (Figure 2A), and (2) rAION-induced vehicle-injected vs. rAION-induced PGJ_2_-treated ONHs (Figure 2B).

A comparison of the involved pathways associated with naïve and vehicle gene sets (Figure 2A) reveals strong upregulation of interferon-related pathway signaling (but not interferons themselves), neuroinflammation and dendritic cell maturation pathway signaling, with moderate upregulation of components associated with both the Th1 and Th2 pathways. There is strong downregulation of the G2/M cell cycle checkpoint (=mitotic upregulation), liver-X and retinoic acid (LXR/RXR) pathway components. LXRs are inhibitors/integrators of inflammation [26]. Following PGJ_2_ treatment post-rAION, there is suppression of the neuroinflammation, TLR and TREM-macrophage and -neutrophil signaling pathways, as well as suppression of leukocyte extravasation and Th1 (classical inflammatory pathway) signaling, but not Th2 (alternative inflammatory pathway). The decrease in NF-κB signaling confirms PGJ_2_’s known role in suppression of this system. Only iCOS-iCOSL pathway signaling, which relates to T-cell activation and germinal center upregulation, was upregulated after rAION following PGJ_2_ treatment (Figure 2).

Because of the ambiguity associated with pathway analysis, we also compared individual inflammation-related genes, including extrinsic macrophage-associated classical (M1) and alternative activation (M2)-associated genes, in the naïve, rAION-induced vehicle- and PGJ_2_-treated animal groups. Il1b and Socs3 were elevated in both vehicle- and PGJ_2_-treated animals, but osteopontin, IL6 and IL1b were reduced compared with vehicle. Il12a levels were similar in all groups. M2-associated cytokines Arg1 and Socs2 were unchanged in all three groups, while the resistin gene (Retn) was slightly elevated in vehicle-treated animals, compared with PGJ_2_-treated animals. The M2-associated resistin-like *beta* gene (Retnlb/FIZZ1) was not detected at all. Expression of the M2-associated gene perioxisomal proliferator-associated receptor *gamma* (Pparg) was elevated 2 fold in rAION-induced, vehicle-injected animals compared with the mean value from naïve animals, and PGJ_2_ treatment reduced this expression to baseline. Thus, PGJ_2_ treatment did not selectively upregulate M2-macrophage responses to any discernable degree.

**Figure 3:**
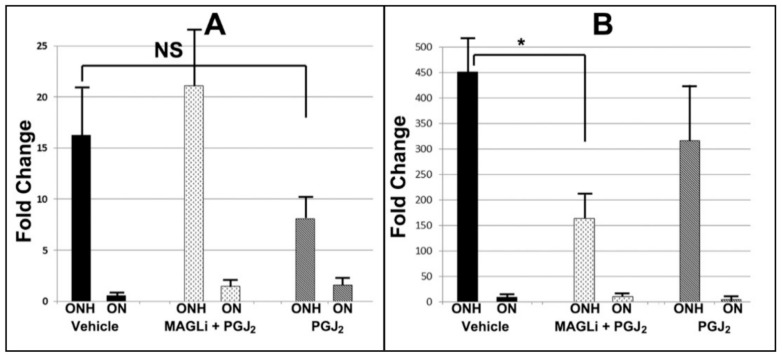
qPCR confirmation of inflammatory gene expression: (**A**) IL6 and (**B**) osteopontin. * *p* < 0.05, ns: nonsignificant.

PGJ_2_ treatment reduced pan-inflammatory gene markers osteopontin (spp1) (Figure 3), Schlafen-3 (slfn4), the Il1 receptor antagonist (Il1rn) and monocyte chemoattractive protein-1 (mcp1-ccl2). Although PGJ_2_ was previously reported to increase expression of Nrf2-related genes, we only detected slightly increased levels of the Nrf2-activated gene NQ01 (nq01) in PGJ_2_-treated animals compared with either vehicle or naïve animals, while heme oxygenase 1 (hmox1) was elevated in both vehicle- and PGJ_2_-treated animals.

Additional individual analyses identified cytokines and their receptors (*n* = 129) (Supplemental Table S1). We evaluated interleukins, chemokines, Toll-like receptors (TLRs) and cytokine-associated suppressors (SOCS) genes, as well as TNFα and TGFβ-mRNAs. 7/38 mRNAs for identified interleukin genes significantly increased expression after rAION induction (Table 4). A list of selected interleukins (significantly increased expression in vehicle vs. naïve) is shown seen in Table 4.

We identified 16/53 chemokines that were expressed at > 2-fold difference between vehicle and mean naïve. Only four (Ccr8, Cxcl10, Ccl22 and Cxcl6) were significantly suppressed by PGJ_2_ treatment. These are seen in Table 5. A comparison of ONH with retina expression revealed that only Ccl3 was upregulated in both ischemic ONH and retina (Table 4, in bold), while retina upregulated Cxcl2, Ccl24 and Cxcl1, and there was no corresponding upregulation in ischemic ONH.

### 3.3. Comparison of ONH Inflammatory Gene Expression with Inflammation in Naïve and Ischemic Retina

Comparing gene expression changes in ischemic ONH and ischemic retina is problematic, since the rapid loss of neurons in ischemic retina will skew interpretation. However, a study of gene expression after induction of temporary retinal ischemic stress (ischemic preconditioning) against a sham population has been previously performed [19]. We compared inflammatory gene expression from this retinal ischemia-sham dataset against inflammatory gene expression changes following rAION induction for similarities. Of retinal genes differentially expressed between 9- and 4-fold following ischemic stress, only 2 (IL6 and IL11) were found to be differentially expressed in the ONH following rAION induction. An inflammation-related gene (osteopontin: Spp1/Optn) was also found to have dramatic changes in expression following rAION induction, and both IL6 and Spp1 expression were differentially affected by PGJ_2_- and combinatorial (PGJ_2_ + KML29) treatment. However, while Spp1 was differentially upregulated in the retina after ischemic stress, there was no significant upregulation of IL6. Two matrix metalloproteinase genes (MMP9 and MMP7) were also differentially expressed (elevated) in both tissues after ischemic stress. Thus, the ischemic stress response patterns of the two neural-ocular tissues appear markedly different, despite some overlaps.

### 3.4. qPCR Confirmation of ONH Gene Expression: Comparison of Vehicle, PGJ_2_ and Combinatorial Treatment

Three animals/group were treated with either vehicle, PGJ_2_ alone or a combination of PGJ_2_ and the MAGL-inhibitor KML29. Three days post-induction, ONH and ON were isolated from uninduced and induced eyes and individual cDNA preparations generated and gene expression evaluated for both IL6 and Spp1. The patterns of expression for these two inflammation-related genes closely matched the expression patterns seen in our deep sequencing experiments (Figure 3).

Total RNA was isolated from ONHs (*n* = 3/rAION + treatment), distal ONs (*n* = 3/rAION + treatment) and tissue from the contralateral uninduced eyes and single-stranded cDNA was prepared from each sample. Gene-specific primers for IL6 and osteopontin were used for qPCR in individual analyses and compared against cyclophilin B (ppib) using the dCT method. Comparison was generated using data (ddCT) obtained from the uninduced side of each animal. rAION induction resulted in a >16-fold increase in IL6 and a 450-fold increase in osteopontin, compared with the contralateral (uninduced) side. PGJ_2_ treatment reduced expression of IL6 by nearly ½ compared with vehicle (16.23 vs. 8.15) but the KML20+ PGJ_2_ combination did not reduce ONH-Il6 expression at all 1/3 (16.26 ± 4.67 vs. 21.16 ± 5.77 sem). The PGJ_2_ treatment difference was nonsignificant (*p* > 0.05). The combination of PGJ_2_+ KML29 reduced osteopontin mRNA expression by 4-fold (450.78 ± 74.45 vs. 164.13 ± 48.57 sem; *p* = 0.02; Mann–Whitney U test), while PGJ_2_ alone reduced osteopontin expression nonsignificantly (ns) (450.78 ± 74.45 vs. 316.61 ± 114.46 sem fold; *p* > 0.05).

ONH-IL6 mRNA levels in vehicle-injected rAION-induced tissues increased 13.5 fold compared with uninduced tissue, while the relative induction in the ON was negligible (Figure 3A; ON). PGJ_2_ treatment reduced IL6 expression by half (16.23 vs. 8.15), although this did not reach significance, likely due to the small number of animals. In contrast, combinatorial treatment nonsignificantly increased expression compared with vehicle (16.23 vs. 21.16). Osteopontin increased 450 fold in the vehicle-treated/ONH-induced animals, vs. 156 fold for PGJ_2_ +KML29-treated animals (significant; *p* < 0.05; indicated by an asterisk (*)), while PGJ_2_ treatment alone reduced osteopontin expression ~ 33% compared with vehicle (451 fold vehicle vs. 317 fold PGJ_2_ treatment over naïve expression).

### 3.5. Effects of Single and Combinatorial Treatment on Optic Nerve Edema

**Figure 4:**
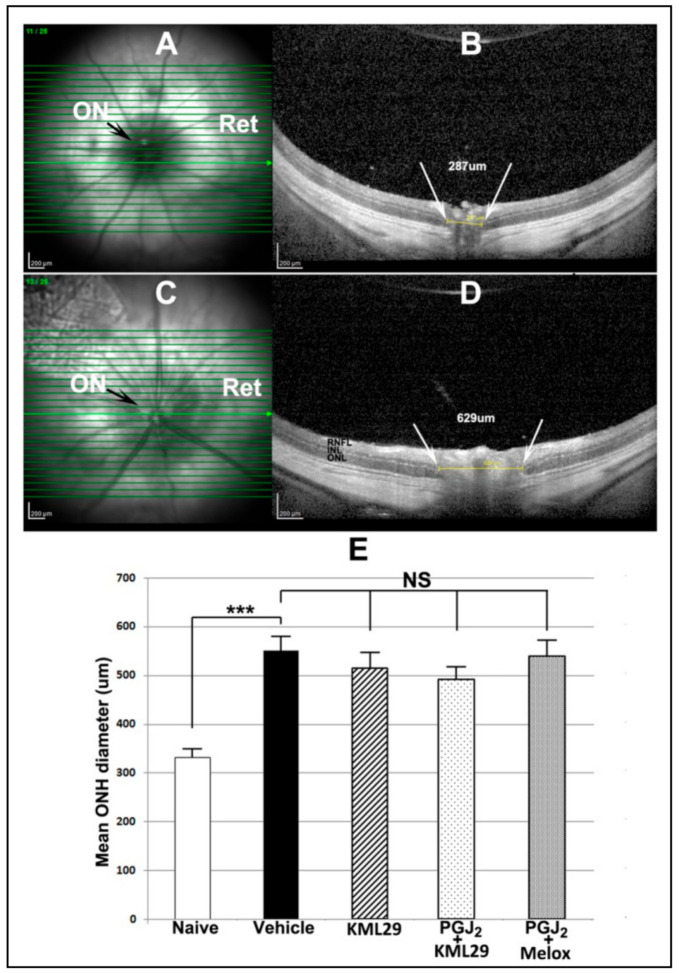
SD-OCT-ONH cross-sectional comparison and mean values for different treatment groups. (**A**,**C**): *en face* views of the optic disk in A) naïve (uninduced) and C) induced vehicle--treated eye. The uninduced eye in A shows a small, dark optic disk (ON), while the induced eye in C has a swollen, edematous disk (ON). (**B**,**D**): Cross-sectional views of the same optic disks seen in A and C, respectively. B. The uninduced eye has a relatively narrow ONH cross-sectional diameter (287 um) as measured between the two edges of the inner nuclear layer (INL) (shown as white arrows in B) in this particular slice. D. The induced eye in shows ONH edema with expansion of the INL–INL distance. Mean ONH diameter is defined as the mean of the three slices with the largest INL–INL distance. (**E**): Mean ONH diameters of naïve (white bar), vehicle (black bar), KML29 alone (MAGL inhibitor) (cross-hatched bar), PGJ_2_ and KML29 combination (light speckle), and PGJ_2_ and meloxicam (Melox) combination. There is a significant expansion of the mean ONH diameter following induction (331.3 ± 8.7 um naïve (*n* = 11) vs. 550.5 ± 22.3 um vehicle (*n* = 11) (sem); (***; significant > *p* < 0.001) *p* = 0.0002; Mann–Whitney two-tailed test). Comparisons between all induced groups were nonsignificant (NS) (*p* > 0.05).

rAION induction results in ONH edema that can be quantified using the Heidelberg SD-OCT, by placing a plano-convex contact lens on the rodent eye and imaging through the retina (Figure 4A–C). The naïve ONH is dark in en face view (Figure 4A), and a single slice diameter in this animal was 287 um (Figure 4B). The mean distance between the two leaves of the inner nuclear layer of the retina (INL–INL distance) in this group of animals was 331 ± 8.74 um (sem). In contrast, post-induction ONH edema is detectable en face by a lightening of the ON shadow (Figure 4C), and expansion of the INL–INL diameter (Figure 4D).

The mean INL–INL ON distance in naïve eyes was 331.3 ± 8.7 μm (sem) (Figure 4E, white bar). The INL–INL mean distance expanded in animals 2 days post-induction to 550.5 ± 22.3 μm sem (Figure 4E, black bar), consistent with ONH edema. This difference is significant (*p* < 0.002). The combination of the MAGL inhibitor (KML29) + PGJ_2_ gave a trend toward reduced ONH edema compared with vehicle: 492.4 ± 17.2 μm sem vs. 550.5 ± 17.2 um (sem), but this was not significant (*p* = 0.068; two-tailed t-test). Combining PGJ_2_ + the general COX inhibitor meloxicam also resulted in nonsignificant edema reduction (539.5 ± 24 μm vs. 550.5 ± 22.3 um (vehicle) (*p* = 0.767; Mann–Whitney two-tailed test). Finally, KML29 alone did not significantly reduce ONH edema compared with vehicle (550.5 ± 22.3 μm vs. 515.2 ± 24.7 μm sem; *p* = 0.328).

### 3.6. Combining either PG Synthesis or COX Inhibitor with PGJ_2_ Does Not Improve RGC Survival Following rAION

**Figure 5:**
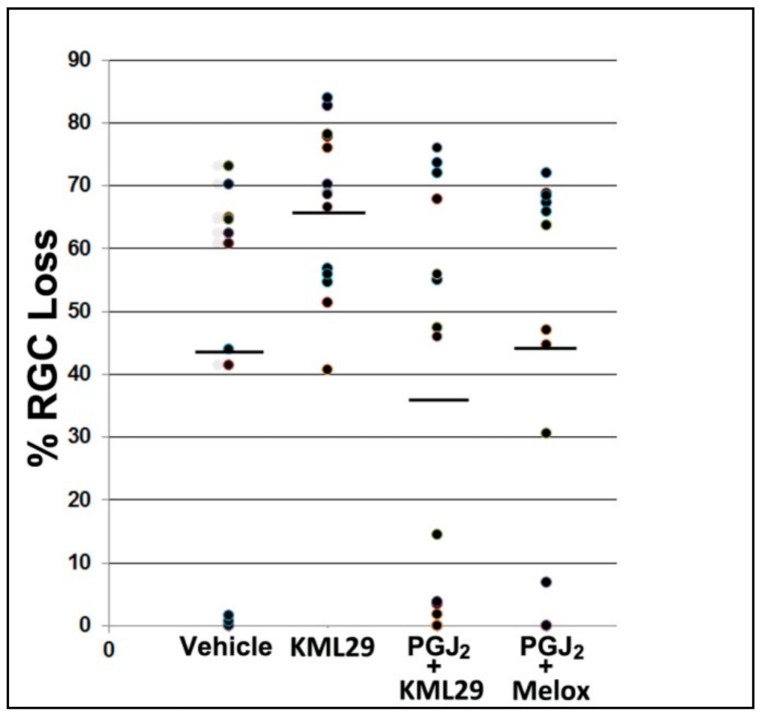
Stereological analysis of RGC loss following rAION induction and treatment. Scatter plot results of Brn3a(+) flat-mounted RGCs 30 d post-rAION induction. Each point represents the RGC loss from one animal compared with the contralateral (uninduced) eye (=100%). Mean RGC loss in the vehicle group was 44.0 ± 9.8% sem. KML29 treatment alone (no PGJ_2_ coadministration) resulted in a statistically significant decrease in RGC survival (66.6 ± 3.7% sem; two-tailed *t*-test; *p* = 0.0275). There was no statistically significant improvement in RGC survival with PGJ_2_ + KML29 (mean 36.6 ± 7.9 sem (*n* = 16) vs. vehicle; 44.0 ± 9.8 sem (*n* = 11); two-tailed t-test; *p* = 0.5651), or with PGJ_2_ + the COX inhibitor meloxicam (Melox) (44.6 ± 9.0 sem (*n* = 11) vs. vehicle, 44.0 ± 9.8 sem; Mann–Whitney one-tailed test; *p* = 0.4286), when compared with vehicle alone.

Isolated PGJ_2_ treatment has been shown to reduce ONH edema post-rAION and improve RGC survival [27,28,29]. RGC stereological quantification 30 post-rAION induction revealed that vehicle administration alone resulted in an RGC loss of 44.0 ± 9% sem (Figure 5, vehicle), while treatment with KML29 alone actually increased RGC loss (66.6 ± 3.7% sem) (Figure 5, KML29). This difference was significant (two-tailed t-test; *p* = 0.0275). In contrast, co-administration of PGJ_2_ with the MAGL inhibitor KML29 generated a nonsignificant decrease in RGC loss compared with vehicle (36.6 ± 7.9% sem) (Figure 5, PGJ_2_ + KML29), and PGJ_2_ + meloxicam yielded RGC losses comparable with vehicle alone (44.6 ± 9.0% sem) (Figure 5, PGJ_2_ + Melox). Thus, isolated KML29 administration actually increased RGC loss, while the combination of either KML29 or meloxicam and PGJ_2_ did not significantly reduce RGC loss below that of vehicle alone.

### 3.7. The MAGL Inhibitor KML29 Does Not Suppress Retinal or ONH PGE_2_ Synthesis

**Figure 6:**
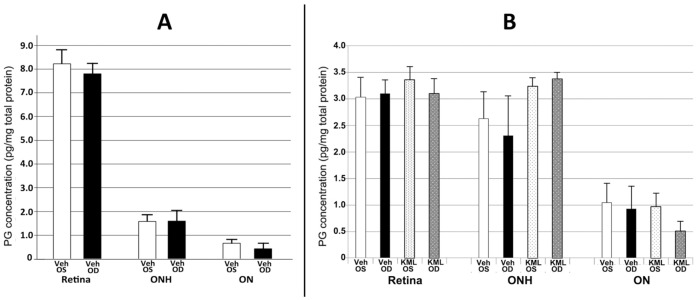
PGJ_2_ and PGE_2_ levels in the retina, optic nerve head (ONH) and optic nerve, and the effects of KML29 treatment (*n* = 3) on PGE_2_. White and black bars represent results from naïve (*n* = 3) and vehicle-injected, 1d post-rAION (*n* = 3) induced eyes, respectively. (**A**) PGJ_2_ levels in retina, ONH and ON. Naïve (Veh OS) retinal PGJ_2_ levels are considerably higher (8.22 ± 0.51 (sem) than those of ONH (1.58 ± 0.24 (sem) or ON (0.66 ± 0.06 (sem), respectively, and there is little change 1d after rAION induction (Veh OD). (**B**) PGE_2_ levels in retina, ONH and ON and the effects of KML29. PGE_2_ levels in the retina are similar in both naïve (Veh OS) and induced (Veh OD) conditions and KML29 does not significantly alter intraretinal expression at one day post-induction (KML OD). KML OS represents the contralateral (uninduced) ocular tissues. PGE_2_- expression is slightly less in ONH, and KML29 slightly upregulates PGE_2_ expression. PGE_2_ expression is ~2-fold less in the distal ON than in the ONH, and neither rAION nor KML29 significantly affect PGE_2_ expression in any tissue. Units are in pg/mg protein.

High levels of PG synthetic gene mRNAs and their proteins do not automatically translate to metabolite levels. PGE_2_ is expressed in a number of CNS tissues following ischemic stress response [30], but PG levels in the resting or ischemic ONH were unknown. Because of the surprising results we obtained from our combinatorial rAION treatment study, we used ELISA to evaluate PGJ_2_- and PGE_2_ levels in tissues from both rAION-induced and uninduced retina, ONH, and ON (Figure 6A,B), and KML29′s ability to reduce PGE_2_ levels in these tissues when treated for the first 24 h following induction (Figure 6B).

Naïve retinal PGJ_2_ levels (8.22 ± 0.51 pg/mg tissue) measured by ELISA are considerably higher than that in the ONH and ON (1.58 ± 0.24 and 0.66 ± 0.06 pg/mg tissue, respectively) (Figure 6A), and rAION induction did not noticeably alter PGJ levels in either retina or ON (Figure 6A: compare black and white bars in each tissue). In contrast, retinal PGE_2_ levels are considerably lower than PGJ_2_ in uninduced and rAION-induced retina (~3.0 pg/mg tissue; Figure 6B; retina, black and white bars). PGE_2_ levels are similar in both retina and ONH in uninduced tissue retinal PGE_2_ expression did not appreciably change when KML29 was administered for the 24 h period prior to tissue collection (Figure 6B: retina, compare vehicle with rAION-induced bars). PGE_2_ is expressed at similar levels in the naïve retina (~3.1 pg/mg tissue) and ONH (2.65 pg/mg tissue) and ON (1.05 pg/mg tissue). One day post-rAION induction, no changes were seen in PGE_2_ expression levels in either the retina or ONH with naïve (Figure 6B; compare retina and ONH). Interestingly, KML29 actually elevated ONH PGE_2_ levels slightly in both rAION-induced and uninduced ONH, compared with vehicle controls (Figure 6B: ONH, compare vehicle- and KML29-treated tissue). Thus, the product levels of PGJ_2_ and PGE_2_ in the retina, ONH and ON bear no relationship whatsoever to the either the mRNA levels of their parent synthetic genes, and the MAGL inhibitor KML29 used in many experiments failed to exert an appreciable effect on PG synthesis, despite published reports of effects in other CNS regions.

## 4. Discussion

There is strong upregulation of inflammatory gene responses in the ONH following rAION induction, and these responses are modified after PGJ_2_ treatment. A large number of inflammatory modulating genes (cytokines, chemokines, Toll-like receptors (TLR’s) are expressed in the naïve ONH, and ischemic ONH induction results only in subtle changes in subsets of these genes 3d post-rAION induction, suggesting that only a limited number of inflammatory response genes may be associated with the early changes seen in our rodent model of ischemic optic neuropathy. However, pathway analysis suggests that these responses converge to strongly influence neuroinflammatory and Th1-associated signaling, as well as upregulation of TLR-related activity. PGJ_2_ treatment suppresses inflammation, but does not upregulate the Th2-alternative inflammatory (eg neuroprotective) response. The ONH’s pattern of response is distinct from, yet overlapping with retina responses.

The most robust cytokine responses were identified as IL6 (ratio of vehicle/naïve (V/N) = 1.66) and IL27 (V/N = 1.30). Neither IL27 nor IL6 were associated with retinal ischemia, unlike that seen in ONH ischemia. Of the top 7 interleukins that changed more than two-fold (IL-6, -27, -24, -10, -3, -1b and -12b), only IL1b showed significant elevation in both the retina ischemia model and ONH in the rAION model. Of seven suppressor of cytokines (SOCS) genes, only SOCS3 showed a pattern of increase that was significantly different than retina (ONH increase). One cause for the difference in the expression pattern differences between the two tissues (ONH and retina) may be the relative difference in reliance on signals associated with microglial activation, which is prominent at this time in the retinal ischemic model, as compared with the ONH ischemic model [28,29].

A larger chemokine subset significantly altered their expression after rAION induction (16/53 identified genes), and the chemokines with the most robust upregulation were Ccl17 (V/N 1.46); Ccl9 (V/N 1.41) and Cx3Cr1 (V/N 1.36). Interestingly, the overall patterns of many chemokines were similar in both the retinal ischemia and rAION models. Almost no cytokines or chemokines declined after rAION. Only a subset of this response group was directly affected by PGJ_2_ administration (including IL6 and IL10, implying that more potent, selective therapeutics are needed to effectively modulate post-ischemic inflammatory responses in the treatment of optic nerve disease.

Osteopontin is a cytokine that can upregulate both expression of Ifn-*gamma* and Il12 [31]. While no expression of Ifn-gamma was detected, there was upregulation of Il12b in rAION-ONH. Additionally, despite the inability of the combinatorial treatment to suppress either PGE_2_ (or PGJ_2_) levels, the combination of KML29 and PGJ_2_ suppressed osteopontin more strongly than PGJ_2_ alone, suggesting that KML29 has other biological effects than as an AA synthesis suppressant. This may be in part responsible for its reported ability to alter CNS function [20,32].

Although the combination of the MAGL inhibitor KML29 and PGJ_2_ reduced ONH edema, compared with vehicle (492.4 ± 17.2 vs. 550.5 ± 22.3), the reduction was not significant (*p* = 0.1178; two-tailed *t* test) (Figure 4). Combining PGJ_2_ + meloxicam or administering KML29 alone was even less effective in reducing ONH edema (539.5 ± 24.0 and 515.2 ± 24.7 um., respectively). We have previously shown that PGJ_2_ treatment reduces ONH edema in our rAION and pNAION models [23,33]. The fact that addition of either a MAGL or COX inhibitor failed to potentiate PGJ_2_’s edema-reducing capability suggests that this failure may result from the inability of either agent to suppress PG synthesis in the ONH.

Similar to the results seen for ONH edema, adding KML29 or the COX inhibitor meloxicam to PGJ_2_ failed to improve RGC survival to levels greater than PGJ_2_ alone (Figure 5), compared with previous studies [23,27]. There was a trend towards RGC preservation in the KML29 + PGJ_2_ treatment group, compared with vehicle, but this was not statistically significant (*p* = 0.56). Interestingly, administration of KML29 by itself resulted in a statistically significant increase in RGC loss, compared with vehicle (two-tailed *t*-test; *p* = 0.0275). Thus, KML29 may enhance RGC loss, independent from any effect on either PGJ_2_ or PGE_2_ synthesis. Indeed the combination of the two drugs may actually block PGJ_2_’s full neuroprotective effect. This treatment dichotomy was partly the reason for wanting to understand both the ONH gene expression patterns that occur following rAION and treatment with PGJ_2_, and the actual production of the target prostaglandins.

Our deep sequencing data reveal that PGD_2_ and its metabolite PGJ_2_ are likely the primary anti-inflammatory PGs, since neither PGI nor PGF synthases were detected in the ONH. All three PGE_2_ synthases (cytoplasmic, membrane) were also detected. While the gene expression level of PGD_2_ synthase (PTGDS; which gives rise to PGJ_2_) is ~200-fold greater than highest expressing isoform of PGE_2_ synthase (PTGES2), the ELISA-measured expression of ONH-PGJ_2_ and PGE_2_ levels were equivalent in naïve- and rAION-induced vehicle-injected, and rAION-induced tissues, suggesting that PG modulation in ocular tissues is complex and unique to each region. KML29 actually increased PGE_2_ levels in the ONH, although this trend was nonsignificant. Interestingly, PGE_2_ expression levels are similar between retina and ONH (~3.5 pg/mg tissue protein), but the relative mRNA expression of levels of the proteins giving rise to PGE_2_ is considerably higher in the ONH than in the retina (see Table 2). PTGDS mRNA expression is also much greater in the ONH and ON than in the retina model in either sham- or ischemic conditions (see Table 2), but despite this difference, PGJ_2_ levels are considerably higher in the retina than in the ONH under any measured conditions (~8.0 pg/mg tissue for retina vs. ~1.7 pg/mg tissue for ONH). It also reveals that the product levels of PGJ_2_ and PGE_2_ in the retina, ONH and ON bear no relationship whatsoever to the either of the mRNA levels of their parent synthetic genes. PG synthetic genes in the ON and ONH may have other functions than simply generating PGs.

An important question remains: Why does a reagent that suppresses 99% of the MAGL activity have no effect on PG synthesis, since it has been reported to do so in other rodent tissues? There are at least two possible answers. First, while KML29 penetrates the majority of the CNS, including brain and spinal cord, there may be reduced penetration of the compound into the retina or optic nerve, or these tissues may have a greater reliance on non-MAGL-PG production. For example, KML29 was unable to reduce PGD_2_ levels in spinal cord, despite the cord exhibiting the highest basal levels of PGD_2_ [22]. Similarly KML29 was unable to decrease PGE_2_ in brown fat. This effect may also be due to alternative PG production by multiple isoforms of PLA_2_. Our deep sequencing analysis reveals that ONH expresses nearly 20- and 10-fold greater mRNA levels of a two cytoplasmic PLA_2_ isoforms (PLA2g7 and PLA2g16; Table 3) than MAGL, and the retina expresses 21–22-fold more of the PLA_2_ isoform PLA2g16 than MGLL/MAGL, suggesting that both the ONH and retina are capable of overcoming MGLL/MAGL inhibition to generate sufficient prostaglandins.

Our results demonstrate that post-ischemic changes in the ONH are complex and far-ranging, with a response pattern considerably different than that seen in the ischemic retina, and which may help explain the differential response to treatments for ischemia in both tissues. It also provides a caveat: potentiating the suppressive effects of inflammatory pathways, while appearing straightforward, is prone to over interpretation and must be carefully scrutinized, with specific control experiments to confirm that there is definite suppression of the proposed pathways; this is seldom performed. The confusing contradictory results and conclusions reported by multiple labs and in multiple treatment trials is undoubtedly associated with the lack of internal analyses. The use of appropriate, well-thought-out internal controls, in the model of interest, must be performed to confirm that results seen are relevant to human disease treatment.

## Figures and Tables

**Table 1:** List of genes and primers used for qPCR. Common name is shown in the first column, the NCBI accession number is shown in the second column. The nucleotide sequence (5′-3′ direction) of each primer pair is shown in the third column, and the primer position (forward/reverse) is indicated in the fourth column for each primer.

Name	Accession #	Sequence (5′-3′)	Direction
Osteopontin	M14656.1	GAGGAGAAGGCGCATTACAG ATGGCTTTCATTGGAGTTGC	forward reverse
IL6	NM_012589.2	CGAGCCCACCAGGAACGAAAGTC TCAGTCCCAAGAAGGCAACTGGCT	forward reverse
Cyclophilin	NM_022536.1	GCTGAAGCACTATGGGCCCGG ACCTTCCCGTACCACATCCATGCCT	forward reverse
Β-Actin	NM_031144.2	TGACGGTCAGGTCATCACTATC GGCATAGAGGTCTTTACGGATG	forward reverse

**Table 2:** Normalized mean relative expression of selected prostaglandin-synthetic and receptor-associated genes in rat ONH and retina. Illumina sequence data for ONH are based on Deseq2-normalized (log_2_-fold relative levels) expressed in ONH RNA from individual naïve, mean naïve (*n* = 5), rAION-vehicle-induced and rAION-PGJ_2_-treated animals, using a cutoff of <3.4. Normalization is based on Trimmed Mean Values (TMM) and normalized by EdgeR-cpm function with a cutoff of 2. Naïve retina (retina-N) and ischemic retina (retina-Isch) are from [19] and expressed in counts per million (CPM). The ratio of expression in ischemic retina/sham retina values are shown in the last column. The main CNS-expressed form of prostaglandin D2 synthetic gene (PTGDS) which gives rise to PGJ_2_ is expressed at extremely high levels in ONH, and in moderately high levels in retina, while the gene for microsomal PGE_2_ synthesis (PTGES2) was higher in ONH than cytosolic PGE_2_ synthesis (PTGES3), while this pattern was reversed in retina. There was no ONH expression of synthetic genes for either PGF_2_ or PGI (prostacyclin). Genes for downstream prostaglandin H synthesis from arachidonic acid (COX1/PTGS1 and COX2/PTGS2) were expressed at moderately high levels in ONH, but at extremely low levels in the retina in both naïve and ischemic conditions. NF: not found.

Gene Symbol	Gene Name	ONH-1	ONH-2	ONH-3	ONH-4	ONH-5	Mean Naïve	Veh-rAION	PGJ_2_-rAION	Veh/Naïve	Veh/PGJ_2_	Ret-Sham (CPM)	Ret-Isch (CPM)	Ret-Isch/Ret-Sham
Ptgds	prostaglandin D2 synthase	15.98822	16.79986	18.33136	16.76294	17.63618	17.54915	17.10371	16.80383	1.02604	1.04435	323.16894	255.03599	0.78917
Hpgds	hematopoietic prostaglandin D synthase	5.56447	5.58306	6.58374	5.64267	5.48511	6.28145	5.77181	5.81564	1.08830	1.08010	0.85582	0.67756	0.79171
Ptges	prostaglandin E synthase	7.02457	6.58184	7.60527	6.82481	7.68730	7.14567	7.14476	7.08125	1.00013	1.00910	1.25531	18.48897	14.72862
Ptges2	prostaglandin E synthase 2	9.96166	9.70580	9.48162	9.83855	8.73850	9.24443	9.54522	9.58994	0.96849	0.96397	33.61580	37.41490	1.11302
Ptges3	prostaglandin E synthase 3-cytosolic	7.67189	7.46936	8.18863	7.39734	8.40670	7.97795	7.82678	8.16406	1.01931	0.97720	404.75993	371.05767	0.91674
Ptges3l1	prostaglandin E synthase 3-like 1	8.35512	7.86037	7.92716	7.59559	7.68333	7.47952	7.88431	7.20205	0.94866	1.03853	34.94840	27.50432	0.78700
Ptges3l	prostaglandin E synthase 3 like	7.67189	7.46936	8.18863	7.39734	8.40670	7.97795	7.82678	8.16406	1.01931	0.97720	NF	NF	NF
Ptgs1/Cox1	prostaglandin-endoperoxide synthase 1	9.76256	9.44712	11.15517	9.28240	9.87118	10.00541	9.90368	9.79640	1.01027	1.02134	0.45581	5.75307	12.62153
Ptgs2/Cox2	prostaglandin-endoperoxide synthase 2	7.22716	7.34416	7.54500	8.28609	9.78677	9.66859	8.03784	9.10447	1.20288	1.06196	0.45155	1.88256	4.16913
Ptgfr	prostaglandin F receptor	12.25038	12.60656	11.73813	12.51715	12.54528	13.07791	12.33150	13.09570	1.06053	0.99864	0	1.99707	0
Ptgr2	prostaglandin reductase 2	12.75745	12.36159	12.25462	12.05000	11.79029	11.52081	12.24279	11.10647	0.94103	1.03731	5.63482	41.96967	7.44827
Ptgdrl	prostaglandin D2 receptor-like	6.14142	6.50112	7.51399	6.14764	7.29350	6.94778	6.71953	6.65179	1.03397	1.04450	NF	NF	NF
Ptgdr2	prostaglandin D2 receptor 2	5.32453	5.97640	6.11997	6.27199	7.58084	6.83036	6.25474	7.13510	1.09203	0.95729	NF	NF	NF
Ptger1	prostaglandin E receptor 1	5.67510	6.13244	6.52818	6.95166	6.01387	6.94449	6.26025	6.78540	1.10930	1.02344	0	0.29343	0
Ptger2	prostaglandin E receptor 2	5.93095	5.54944	7.38904	5.90408	7.01148	6.93787	6.35700	6.28318	1.09137	1.10420	0	0	0
Ptger3	prostaglandin E receptor 3	5.56447	5.12102	6.11997	4.69013	3.66247	5.58335	5.03161	5.72828	1.10965	0.97470	NF	NF	NF
Ptger4	prostaglandin E receptor 4	5.67510	5.04621	3.66247	5.83632	5.36677	6.78596	5.11737	6.81983	1.32606	0.99503	0	0.207717	0
Ptgfrn	prostaglandin F2 receptor inhibitor	5.56447	5.12102	6.11997	4.69013	3.66247	5.58335	5.03161	5.72828	1.10965	0.97470	73.28975	66.36605	0.90553
Ptgir	prostaglandin I2 receptor	5.85502	6.32898	5.98393	6.38106	7.36330	7.69776	6.38246	7.76910	1.20608	0.99082	0	0.88028	0
Ptgr1	prostaglandin reductase 1	8.21237	8.89242	9.85389	7.87723	9.15406	8.89142	8.79800	8.93364	1.01062	0.99527	32.70928	36.26502	1.10871

**Table 3:** List of arachidonic acid synthetic genes: phospholipase A2 genes and MAGL/MGLL. Illumina sequence data for ONH are based on Deseq2-normalized (log_2_-fold relative levels) expressed in ONH RNA from naïve, rAION-vehicle-induced and rAION-PGJ_2_-treated animals, using a cutoff of <3.4. Normalization is based on Trimmed Mean Values (TMM) and normalized by EdgeR-cpm function with a cutoff of 2. Naïve retina (retina-N) and ischemic retina (retina-Isch) are from [19] and expressed in counts per million (CPM). The ratio of expression in ischemic retina/sham retina values are shown in the last column. Gene-mRNA quantification and differential expression can be equated between the two studies (ONH and retina) by comparing the relative expression of both standard housekeeping genes (Beta-Actin (Actb), cyclophilin a ((ppia), cyclophilin b (ppib), glyceraldehyde phosphate dehydrogenase (GAPDH) and PPARγ (pparg), as well as the expression of some of the highest expressing genes in each tissue (MBP in ONH; Rho in retina). NF: not found.

Gene Symbol	Gene Name	ONH-N1	ONH-N2	ONH-N3	ONH-N4	ONH-N5	Mean Naïve	Veh-rAION	PGJ_2_-rAION	Veh/Naïve	Vehicle/PGJ_2_	Ret-Sham (CPM)	Ret-Isch (CPM)	Ret-Isch/Ret/Sham
Pla2g7	phospholipase A2 group VII	15.10933	14.79132	12.94959	14.77261	12.82698	14.089966	13.003512	13.335868	0.9228917	0.975078036	0.35146081	33.993338	0.010339
Pla2g16	phospholipase A2, group XVI	13.63818	12.91624	13.40673	12.66619	12.365	12.998467	12.1254154	12.018962	0.9328343	1.008857111	235.236921	297.265176	0.791337
Pla2g6	phospholipase A2 group VI	10.18158	10.04404	10.07626	10.34514	9.786774	10.086759	10.0580887	10.407593	0.9971576	0.966418349	11.0373495	14.7100521	0.750327
Pla2g4a	phospholipase A2 group IVA	8.836592	8.972135	9.337722	9.232697	10.36593	9.3490153	10.1824257	9.5777503	1.0891442	1.063133338	9.53095284	20.7441067	0.459454
Pla2g15	phospholipase A2, group XV	9.021099	9.191856	9.603836	9.060801	9.575812	9.2906808	9.86609055	9.9238729	1.0619341	0.994177444	30.7126327	33.3418939	0.921142
Pla2g12a	phospholipase A2, group XIIA	9.676339	9.435444	9.30206	9.447172	9.004778	9.3731584	9.41553769	9.7066862	1.0045213	0.970005369	5.15867823	4.19978952	1.228318
Pla2g3	phospholipase A2, group III	8.88697	8.229182	6.696327	8.932058	6.585697	7.8660468	7.30830417	7.5213669	0.9290949	0.97167234	NF	NF	NF
Pla2g2c	phospholipase A2, group IIC	7.839402	7.646561	6.955483	7.387974	6.77704	7.321292	6.70783566	7.2020536	0.9162093	0.93137819	4.15672828	2.80687237	1.480911
Pla2g4b	phospholipase A2 group IVB	7.73606	8.229182	8.981785	8.117569	8.634831	8.3398853	8.46154825	8.4582903	1.0145881	1.000385178	3.29683962	9.62216053	0.34263
Pla2g5	phospholipase A2, group V	6.704759	8.235359	3.662474	7.034882	6.198245	6.3671437	6.56962921	6.4612068	1.0318016	1.016780527	3.76985453	5.81568605	0.648222
Pla2g2a	phospholipase A2 group IIA	7.158225	6.601056	6.469342	5.929593	6.384064	6.5084562	7.37481481	7.4208545	1.1331128	0.993795911	0.98906215	0.54023288	1.830807
Pla2g2e	phospholipase A2, group IIE	7.240552	7.280109	5.870084	6.790849	6.548524	6.7460235	6.10158817	6.3282454	0.9044718	0.964183242	0	0.27011644	0
Pla2g1b	phospholipase A2 group IB	5.640167	5.121023	3.662474	5.173576	3.662474	4.6519426	5.48358443	4.9921317	1.178773	1.098445474	4.35437038	4.03998796	1.077818
Pla2g4f	phospholipase A2, group IVF	5.906466	5.995318	5.571123	5.891002	5.64408	5.8015978	5.70985447	6.172554	0.9841865	0.925039216	NF	NF	NF
Pla2g2d	phospholipase A2, group IID	6.501938	5.046214	7.169554	5.729765	6.126362	6.1147665	6.48782433	5.8557298	1.0610093	1.107944629	0	1.90726317	0
Pnpla2	patatin-like phospholipase domain containing 2	5.708367	5.995318	5.607421	6.579621	5.584945	5.8951345	6.33600637	6.8198346	1.0747857	0.929055717	20.634368	21.9832379	0.938641
Pla2g10	phospholipase A2, group X	3.662474	3.662474	3.662474	4.690134	3.662474	3.8680059	3.66247389	3.6624739	0.9468636	1	0	1.08409959	0
Pla2r1	phospholipase A2 receptor 1	5.977731	6.193288	6.550772	6.304503	6.548524	6.3149638	6.84479358	6.5347544	1.0839007	1.047444655	67.6342502	74.59747	0.906656
Mgll/MAGL	monoglyceride lipase	10.45186	10.21227	9.67473	10.12171	8.472095	9.7865318	9.61457884	10.15996	0.9824296	0.946320558	99.503189	74.4858603	1.335867
	**Gene expression controls**													
Ppia	peptidylprolyl isomerase A	14.88068	14.21798	15.31686	14.29613	15.27559	14.935534	14.8897368	14.79745	1.0062367	0.996933659	15.5049789	17.7037818	0.8758
Ppib	peptidylprolyl isomerase B	13.29631	12.87667	13.54295	12.97108	13.79579	13.421187	13.3416644	13.296562	1.003392	0.994074854	56.1145906	102.154809	0.549309
Actb	actin, beta	17.01179	16.81788	17.20874	16.96558	17.38982	17.599677	17.4216397	17.078761	1.0200763	0.989884031	631.922522	2276.3337	0.277605
Gapdh	glyceraldehyde-3-phosphate dehydrogenase	14.429	13.95914	14.01805	14.01042	15.03571	15.000118	14.9295525	14.290462	1.0447214	0.995295699	78.0188393	65.4709231	1.191656
Pparg	peroxisome proliferator-activated receptor gamma	5.564472	4.852943	3.662474	5.041199	6.095778	4.9076087	5.89109898	5.0433732	1.1680871	1.200401111	0	1.01533353	0
Mbp	myelin basic protein	18.37768	18.23455	17.80142	18.32891	17.65172	18.175157	17.9969671	18.078857	0.9954704	0.99019598	7.4746679	2.33938774	3.195139
Rho	rhodopsin	6.177695	7.738614	7.482357	8.943992	7.457686	7.8474819	6.45206772	7.5600688	0.8534403	0.822183193	5309.71918	3564.86423	1.489459

**Table 4:** List of interleukins with post-induction elevation > 2 fold (vehicle/naïve). Illumina sequence data for ONH are based on Deseq2-normalized (log_2_-fold relative levels) expressed in ONH RNA from individual naïve, mean naïve (*n* = 5), rAION-vehicle-induced and rAION-PGJ_2_-treated animals, using a cutoff of <3.4. Normalization is based on Trimmed Mean Values (TMM) and normalized by EdgeR-cpm function with a cutoff of 2. Naïve retina (tetina-N) and ischemic retina (retina-Isch) are from [19] and expressed in counts per million (CPM). The ratio of expression in ischemic retina/sham retina values are shown in the last column. The two greatest ratio changes in retinal conditions are shown in bold. The total list is found in Supplemental Data Table S1. NF: not found.

Gene Symbol	Gene Name	ONH-1	ONH-2	ONH-3	ONH-4	ONH-5	Mean Naïve	Veh-rAION	PGJ_2_-rAION	Veh/Naïve	Veh/PGJ_2_	Ret-Sham (CPM)	Ret-Isch (CPM)	Ret-Isch/Ret-Sham
IL6	interleukin 6	3.66247	4.85294	3.66247	4.37461	6.01387	**4.51327**	**7.49741**	6.36064	1.66119	1.17872	0	4.40138	4.4013766
IL27	interleukin 27	4.77202	5.04621	3.66247	4.92983	3.66247	**4.41460**	**5.72260**	5.12828	1.29629	1.11589	0	0	
IL24	interleukin 24	3.66247	3.66247	3.66247	3.66247	3.66247	**3.66247**	**4.69557**	3.66247	1.28208	1.28208	0	0	
IL10	interleukin 10	3.66247	3.66247	3.66247	3.66247	3.66247	**3.66247**	**4.48830**	3.66247	1.22548	1.22548	0	0	
IL3	interleukin3	3.66247	3.66247	3.66247	3.66247	3.66247	**3.66247**	**4.48830**	3.66247	1.22548	1.22548	0	0	
IL1b	interleukin 1 beta	6.35398	5.67498	5.79262	5.62387	7.04861	**6.09881**	**7.43155**	6.55232	1.21852	1.13419	0.761532	370.6914	**486.7708**
IL11	interleukin 12	5.52316	5.73014	7.38336	5.08902	10.45924	**6.83699**	**8.26344**	7.48443	1.20864	1.10408	1.128868	25.52798	**22.613783**

**Table 5:** List of selected chemokines and relative levels expressed in the ONH in naïve, rAION-vehicle-induced and rAION-PGJ_2_-treated animals. Illumina sequence data for ONH are based on Deseq2-normalized (log_2_-fold relative levels) expressed in ONH RNA from individual naïve, mean naïve (*n* = 5), rAION-vehicle-induced and rAION-PGJ_2_-treated animals, using a cutoff of <3.4. Normalization is based on Trimmed Mean Values (TMM) and normalized by EdgeR-cpm function with a cutoff of 2. Naïve retina (retina-N) and ischemic retina (retina-Isch) are from [19] and expressed in counts per million (CPM). The ratio of expression in ischemic retina/sham retina values are shown in the last column. The two greatest ratio changes in retinal conditions are shown in bold. The total list is found in Supplemental Data Table S1. NF: not found. The total list is found in Supplemental Data Table S1.

Gene Symbol	Gene Name	ONH-1	ONH-2	ONH-3	ONH-4	ONH-5	Mean Naïve	Veh-rNAION	PGJ_2_-rNAION	Veh/Naïve	Veh/PGJ_2_	Ret-Sham (CPM)	Ret-Isch (CPM)	Ret-Isch/Ret-Sham
Ccl17	C-C motif chemokine ligand 17	3.66247	3.66247	3.66247	4.78510	5.99643	**4.35379**	**6.34187**	5.47966	1.45663	1.15735	0	0.58685	0
Ccl9	chemokine (C-C motif) ligand 9	3.66247	4.50516	3.66247	4.69013	5.72428	**4.44890**	**6.24965**	4.80497	1.40476	1.30066	0.22664	0.58685	2.589332
Ccl17	C-C motif chemokine ligand 17	3.66247	3.66247	3.66247	4.78510	5.99643	**4.35379**	**6.34187**	5.47966	1.45663	1.15735	0	0.58685	0.00000
Ccl9	chemokine (C-C motif) ligand 9	3.66247	4.50516	3.66247	4.69013	5.72428	**4.44890**	**6.24965**	4.80497	1.40476	1.30066	0.22664	0.58685	2.589332
Cxcl11	C-X-C motif chemokine ligand 11	4.55453	5.24570	3.66247	6.44499	6.62156	**5.30585**	**7.10257**	6.85985	1.33863	1.03538	1.17676	4.84968	4.121221
Cxcl3	chemokine (C-X-C motif) ligand 3	3.66247	3.66247	3.66247	3.66247	6.09578	**4.14913**	**5.50131**	5.32898	1.32589	1.03234	0	1.76055	1.76000
Ccl22	C-C motif chemokine ligand 22	4.91547	4.50516	3.66247	3.66247	6.03093	**4.55530**	**5.94922**	5.06457	1.30600	1.17468	0	0.44014	0.00000
Cxcl10	C-X-C motif chemokine ligand 10	6.91014	6.74324	7.41151	7.34027	8.74746	**7.43052**	**9.62249**	7.72673	1.29499	1.24535	2.30759	6.96402	3.017873
Ccl3	C-C motif chemokine ligand 3	6.10348	5.47653	3.66247	5.72976	7.46226	**5.68690**	**7.36272**	6.85985	1.29468	1.07331	**0.22664**	**113.3327**	**500.0525**
Ccl4	C-C motif chemokine ligand 4	5.11673	5.18679	3.66247	5.21148	7.04250	**5.24399**	**6.75924**	6.41225	1.28895	1.05411	0	7.18892	7.18000
Ccl11	C-C motif chemokine ligand 11	5.97773	5.99532	5.23550	6.27199	8.79818	**6.45574**	**8.03063**	7.15605	1.24395	1.12222	0.98315	0.13551	0.137835
Ccl21	C-C motif chemokine ligand 21	3.66247	3.66247	3.66247	3.66247	5.97860	**4.12570**	**5.06042**	4.90761	1.22656	1.03114	0.64187	0	0.00000
Ccr8	C-C motif chemokine receptor 8	3.66247	3.66247	3.66247	3.66247	3.66247	**3.66247**	**4.48830**	3.66247	1.22548	1.22548	NF	NF	NF
Cxcl17	C-X-C motif chemokine ligand 17	6.19527	5.80569	3.66247	5.83632	5.92253	**5.48446**	**6.66235**	6.27156	1.21477	1.06231	0.70292	0.68646	0.97658
Cxcl6	C-X-C motif chemokine ligand 6	5.26306	4.95893	6.17198	5.39903	6.61271	**5.68114**	**6.80838**	5.89380	1.19842	1.15518	0	6.45535	6.4
Ccl7	C-C motif chemokine ligand 7	6.10348	5.29929	7.40593	5.17358	8.71593	**6.53964**	**7.78204**	6.92987	1.18998	1.12297	0.42791	621.8639	**1453.252**
Cxcl2	C-X-C motif chemokine ligand 2	5.38041	5.34857	3.66247	4.69013	6.71428	5.15917	6.01198	5.83595	1.16530	1.03016	**4.40896**	**408.5915**	**92.67299**
Ccl24	C-C motif chemokine ligand 24	6.72505	6.37803	6.89992	6.51138	7.96817	6.89651	7.86946	6.45161	1.14108	1.21977	**1.24787**	**164.22**	**131.5999**
Cxcl1	C-X-C motif chemokine ligand 1	5.32453	5.43672	6.57287	5.34265	7.33875	6.00310	6.70378	6.47071	1.11672	1.03602	**1.95483**	**817.0636**	**417.9714**

## Data Availability

Data has been archived on Figshare: All raw deep sequencing data is available.

## Supplementary Materials

The following are available online at https://www.mdpi.com/article/10.3390/cells10061440/s1, Table S1: All rat values, Table S2: Inflammatory genes.
